# Developing A Complex Intervention to Integrate Community Paramedics in GP Out-of-Hours Care in Ireland: A Qualitative Study

**DOI:** 10.12688/hrbopenres.14246.1

**Published:** 2026-01-09

**Authors:** Colette Cunningham, Siobhan Masterson, Alan Batt, JD Heffern, Shane Knox, Diarmuid Quinlan, Cathal O'Donnell, Deirdre O'Donnell, Tomas Barry

**Affiliations:** 1 School of Medicine, University College Dublin School of Medicine, Dublin, Leinster, Ireland; 2 National Ambulance Service, Health Service Executive, Dublin, Ireland; 3 Faculty of Medicine Nursing and Health Sciences, Monash University Faculty of Medicine Nursing and Health Sciences, Clayton, Victoria, Australia; 4 Community Paramedicine, International Roundtable on Community Paramedicine, Indigenous Services, Canada, Canada; 5 Irish College of General Practitioners, Dublin, Leinster, Ireland; 6 Centre for Interdisciplinary Research, Innovation and Education in Health Systems, University College Dublin School of Nursing Midwifery and Health Systems, Dublin, Leinster, Ireland; 7 School of Population Health, Royal College of Surgeons in Ireland, Dublin, Leinster, Ireland

**Keywords:** Community Paramedic, General Practitioner, Out-of-Hours Care, Model Development

## Abstract

**Background:**

Significant workforce challenges are evolving in Irish general practice, which limit all aspects of practice, especially out-of-hour care. Community Paramedics can support general practitioner (GP) out-of-hour care and potentially improve the system efficiency in Ireland.

**Methods:**

Qualitative semi-structured interviews were conducted with purposively recruited participants using a schedule informed by the existing research. A thematic analysis was undertaken based on the Braun and Clarke approach and supported by the NVivo software. Themes were developed based on the concept of preliminary key model components. The study was conducted in accordance with Standards for Reporting Qualitative Research.

**Results:**

Comprehensive data analysis generated four major themes: Workforce Management, Synergies in Healthcare, Progressive Futuristic Care, and Safe Practice. These themes formed the basis for the further development of the preliminary key model components.

**Conclusion:**

This study suggests that the integration of Community Paramedics into GP out-of-hour care in Ireland can be successfully launched if workforce planning is carefully considered. The role of the Community Paramedic must be defined, regulated, and governed in conjunction with the National Ambulance Service, the Pre Hospital Emergency Care Council, and the Department of Health in Ireland. Patient care must be underpinned by safe and effective care practices that are continuously audited and evaluated, and the scope of practice must be defined and maintained by each Community Paramedic through a guided educational system that incorporates continuous professional development. GPs can provide support to community paramedics, including the use of information technology, as required. GPs and Community Paramedics can work together to synergize healthcare and provide safe and effective care to patients in the domiciliary setting during out-of-hour hours, thus reducing emergency department attendance and increasing the GP’s capacity to provide face-to-face consultations.

## Introduction

### Slaintecare

In 2017, the Health Service Executive (HSE) and Department of Health in Ireland launched an overarching health policy strategic initiative called “Slaintecare.” Slaintecare aimed to reform Ireland’s healthcare and social care system to respond to the increasing complexity of healthcare needs in the community ^1^. An underpinning principle of Slaintecare is that people remain healthy in their homes and communities for longer. A key objective is to optimize the capacity of primary and community care, thus reducing the incidence of ED (emergency department) or hospital visits. The most recent HSE National Service Plan (2025) highlights that Ireland’s population is currently at 5.3m people, with a life expectancy at birth of approximately 82 years ^2^. Therefore, the growing need for an aging population requires expanded and strengthened primary and community care services to cope with the rising demands. This National Service Plan (2025) outlines how the Irish population should have alternative care pathways, with an orientation towards general practice and primary care in particular ^2^. Despite this, the number of patients attending EDs in Ireland has reached record levels by 2024, amounting to 1.6 million visits ^2^. This prompted the HSE to launch an “Urgent Care and Operational Plan” to support timely and safe access to services for patients across the Irish healthcare system ^3^. While this plan will strive to improve the ED visit rate and reduce pressure on acute hospital services, an unintended effect will be to worsen the pressures faced by General Practitioners (GPs) and primary care services that are already overburdened by their own cohorts of patients. This factor was also expressed in the Irish Medical Council (IMC) (2022) report, where the increased demand for an already understaffed GP system was voiced as a critical concern, with a call for longer-term solutions to the GP shortage as crucial ^4^. The problem is further confounded by the key finding of an Irish College of GPs (ICGP) report (2023) ^5^ which highlighted that more than three-quarters of the 2,500 GP practices in Ireland have now closed their lists to new patients seeking GP services in their communities, with the shortage of GPs in Ireland expected to exceed 1000 by 2025 ^6^.

### General Practitioner workforce deficit

The Irish Health Service Executive (HSE) reported more than one million GP out-of-hour (OOH) visits in 2020. The patient service was run by 15 GP cooperatives (CO-OPS), ^7^. The Irish Medical Council (IMC) 2022 report detailed the acute shortage of GPs in Ireland and called for urgent action to address the problem ^4^. This acute shortage of GPs has affected the delivery of high-quality, timely patient care. Currently, there are 2,333 (52.7%) female GPs and 2,098 (47.3%) male GPs in Ireland, with 595 (13.4%) aged > 65 ^8^. Thus, the shortage will be further exacerbated in the next four–five years when this group retires. Furthermore, there was a significant group of 873 (19.7%) between the ages of 55 and 64 years who will likely retire within the next 10 years. Ultimately, there are only 0.86 GPs in practice per 1,000 people in Ireland; however, the majority report a working week in excess of 48 h ^8^. This not only contravenes the European Working Time Directive (2003), but also contributes significantly to burnout and reduced morale among GPs ^9^. For the majority of GPs who are self-employed in their own practices, the European Working Time Directive does not govern their working hours; therefore, there is a high possibility that GPs work in excess of the recommended weekly hours. This year (2025), the ICGP projected the need for 943-1211 additional GPs in Ireland by 2040 ^10^. A total of 350 medical graduates were enrolled in the GP training in 2025. If this entry continues annually and Ireland retains this GP cohort, it is hoped that the GP shortage will lessen in the future ^10^. However, it remains critically important to urgently address the current acute workforce deficit impact on healthcare in Ireland.

### Community Paramedics

Community paramedics (CPs) are relatively novel cadres of healthcare professionals both internationally and in Ireland ^11^. In Ireland, CPs are registered as Advanced Paramedics (APs) and general paramedics who operate with an expanded scope of practice within ambulance services in community and primary care settings. Their role is to improve access to healthcare and provide preventative care, allowing patients to remain in their own homes as much as possible. Existing evidence suggests that paramedics have a broad scope of practice that could enable them to contribute to general practice and primary care, and that paramedics can free time for GPs to conduct face-to-face consultations, provide a timelier service, and bring additional skills into the general practice setting ^12, 13^.

Rural and isolated communities frequently experience delays or lack of GP access ^14^. This is particularly exacerbated in the GP-OOH setting, as GPs may work autonomously without the added support of practice nurses and advanced practice nurses, who only work “in hours” and not at night or on weekends. A pilot project involving CPs from Ireland, Northern Ireland, and Scotland was conducted under the European Union (EU)-funded Co-Operations and Working Together (CAWT) Health and Social Care Partnership from 2017 to 2020 ^11^. This pilot saw CPs working with GPs to provide primary and preventive care to patients in their homes. They reported a combined 85% decrease in hospital or ED attendances, particularly in the 81–90-year-old category of patients ^11^. Following its success, in 2021, and in conjunction with the University College Cork (UCC), Ireland, the NAS began to educate paramedics at the master’s level with a particular focus on community paramedicine. A small cohort of CPs currently work “in-hours” with GPs under the NAS governance. They do not currently work in GP-OOH services and do not have a fully protected time in general practice. This means that if an acute call comes through the NAS and the CP is the closest advanced paramedic to the site, the call will be redirected to an emergency situation.

The CAWT project highlighted the potential for CPs to operate within a community setting, effectively reducing the need for hospital transfers and lessening the burden of domiciliary visits for GPs. This shift not only frees up GPs to conduct face-to-face consultations but also aligns with the Slaintecare objective of keeping people in their own homes and communities for healthcare, thereby decreasing reliance on GP or ED attendances. However, the core challenge lies in understanding how to integrate CP services optimally into existing primary care pathways. This requires fostering effective interprofessional collaboration between GPs and CPs to integrate care and optimize patient services.

### Purpose of the study

GP-OOH service in Ireland is under increasing pressure ^7, 4^. The role of CPs in Irish General Practice is currently only provided “in hours” and by a small cohort of the workforce ^11^. There is a paucity of literature describing care models that integrate CPs into general practice models or that evaluate the impact of this care model on service outcomes ^15– 17^. This qualitative study aims to establish the key elements of a potential GP-OOH/CP collaborative care initiative by exploring the perspectives of key stakeholders responsible for the design, implementation, and operation of the service model. This study aimed to contribute to the development of key model components that outline how this complex intervention will be expected to lead to its effects and under what conditions; ascertain the key issues and factors that would need to be accounted for in the intervention design; and identify the relevant outcomes to ascertain its impact. The study objective was to uncover in-depth insights from purposively sampled research participants from current GP, CP, and healthcare experts, and to develop an inventory of key care model considerations.

## Methods

### Research rationale

This study aimed to contribute to the development of key model components to theorize how a novel and complex intervention would be expected to lead to its effects and under what conditions. We approached this study from an interpretivist perspective as we wished to explore subjective human experiences and how they may affect the research conclusions ^18^. Qualitative research methods were used to support the exploratory nature of the required data collection. Currently, there is no collaborative GP-OOH and CP model of care in Ireland, and there is a paucity of literature on the role of CP in GP practices, both nationally and internationally. Therefore, this qualitative study can generate new insights into addressing this research problem using a population sample of GPs, CPs, health care managers, and experts. A phenomenological approach was used to collect and analyze the data. This incorporated in-depth questioning of participants’ real-life experiences, memories, and understanding of real events to enable researchers to find true and realistic meanings in the area of investigation ^19, 20^.

### Research team

The research team consisted of various professional collaborators interested in paramedicine, general practice, health systems, and national strategy development. CC and TB were the principal investigators in this study. TB is an academic GP and Associate Professor and a member of the PHECC and has played a leadership role in the development of the national CP regulatory framework. CC is a postdoctoral researcher with experience in qualitative research. CC had no previous work experience in the area of investigation and thus led the data collection and analytic process from an open perspective.

### Research participant recruitment

Purposive sampling identifies individuals who provide experts with rich information about the phenomenon under study ^21^. As GP-OOH collaboration with CPs is not in place in Ireland, it was important to purposively recruit GPs and CPs in the Irish setting, allowing valuable insight into the current working situation. Academic paramedicine experts in Ireland and international experts with experience in GP-OOH and CP collaborations were also sought. Finally, health service leaders in Ireland were invited to participate in a change to the current Irish health care system. Participants were invited from existing professional paramedicine, general practice research, and clinical networks. No advertisements are used. This combination of key stakeholders established an expert group, where opinions and advice would be appraised on the proposed development of a complex intervention to integrate CPs in GP-OOH care.

A systematic review of empirical tests in 2022 ^22^, sought to uncover adequate sample sizes for data saturation in qualitative research. The reviewed studies reached data saturation, with 9–17 participants using interviews to collect data. This study targeted three main groups: GPs, CPs, and academic/health service/paramedic experts. We aimed to recruit at least three participants in each group from Ireland, Northern Ireland, and the United Kingdom, and with a variance of male and female recruits. Ireland, where the care model was piloted, was chosen to recruit participants purposively. Therefore, the current work environment could be explored. The UK and NI were targeted, as there were GP/CP collaborations in place, and gaining the lived experience of such a model in motion was of interest to the authors. Conceptual categories in qualitative research are considered saturated when there are no new insights or information forthcoming from repeated qualitative data collection ^22^. Therefore, if this sample did not allow for the generation of redundant information, further participants would have been invited until data saturation was satisfactorily reached. Table one outlines participants’ professional discipline, gender, and country of work.

A total of 12 participants were enrolled in the study; four GPs, three CPs and four health service/paramedic/academic experts. One of the experts was a CP expert. A total of 58% of the participants were male (n=7) and 42% were female (n=5). Of the sample, 66% were from Ireland (n=8), 25% (n=3) were from the UK, and 8% (n=1) were from Northern Ireland.

### Ethics and consent

Ethical approval was granted by the University College Dublin Health Research Ethics Committee prior to data collection (ref; LS-LR-24-305-Barry).

### Recruitment

Purposively sampled participants were sent a letter of invitation and participant information leaflet. When an interest in participation was received, a digital consent form was sent for recruitment. A digitally signed consent form was received from each participant before the time and date of the interview were agreed upon. All correspondence was digital via a password-protected university laptop to which only CC and TB had access. All consent forms and personal details were stored separately in an encrypted password-protected folder on the university drive system. Each participant was assigned a non-identifiable code to maintain anonymity and protect their confidentiality throughout the study.

### Data collection

The interviews took place and were recorded using the university’s teleconferencing system. Participants were assured of confidentiality when speaking and their consent was reiterated at the beginning of the interviews. It was explained to the participants the format of the interview that would be taken, that they would be voice recorded, and that they could stop the interview at any time during the meeting. Once the interview was completed, the recording was electronically transcribed into a textual document and uploaded to a password-protected NVivo file. After transcription verification and anonymization by CC, recorded interviews were deleted.

A semi-structured interview schedule was developed using existing research as a rationale for questioning, and the study’s overall aims and objectives as its core structure. Participant recruitment began on October 15 ^th^, 2024. Twelve participants were interviewed ( Table 1). The first interview also acted as a pilot upon which, following a joint review by the CC and TB, minor adjustments were made to the interview schedule. CC made reflective notes following each interview to ensure that the question schedule continued to answer the research questions. Following the adjustments in interview one, no new adjustments were made. Each interview was transcribed, verified, and anonymized. Following this, transcripts were immediately coded using NVivo software. Data saturation was deemed satisfactory following these 12 interviews as no new codes or subthemes were generated following the analysis. The participant recruitment phase ended on February 7 ^th^, 2025. The shortest interview time was 18 min, and the longest was 1hr 4m mins long. The average interview time was 36 minutes.

**Table 1.** Participants.

PARTICIPANTS	PROFESSIONAL DISCIPLINE	GENDER	COUNTRY OF WORK
1	GP	Male	Ireland
2	GP	Male	Ireland
3	GP	Female	United Kingdom/Northern Ireland
4	GP	Male	Ireland
5	CP	Female	Ireland
6	CP	Male	Ireland
7	CP	Female	United Kingdom/Northern Ireland
8	Health Service/Paramedic Expert	Male	Ireland
9	Academic/Health Service/Paramedic Expert	Male	United Kingdom/Northern Ireland
10	CP/Academic/Health Service/Paramedic Expert	Female	United Kingdom/Northern Ireland
11	Health Service Expert	Female	Ireland
12	Health Service/Paramedic Expert	Male	Ireland

### Data analysis

The data analysis was conducted using Braun and Clarke’s six-phase thematic analysis framework ^23^. This method allowed the authors to systematically identify, organize, and develop patterns of meaning across interview transcripts. This then ‘made sense’ of the shared experiences of the participants and the commonalities of the topics under investigation. An inductive approach to data analysis was undertaken so that what was “in” the data could be deciphered. The themes derived from the data closely matched the content of the interviews. As recommended by Braun and Clarke, thematic maps were created for each study area. This occurs through the “six-phase process” outlined below.

1. **Familiarising yourself with the data;** each interview was electronically transcribed to a word document. The transcripts were read by CC and “corrected” while listening to each recording. It was then listened to again while the corrected transcripts were read. “Notes of interest” were also annotated in this process as CC became familiar with the data. Following this process, the recording was deleted and the transcript was uploaded to NVivo. It was then read by TB. Reading and re-reading the data allowed the authors to immerse themselves in the content.
2. **Generating initial codes;** All documents were coded using NVivo. A coding box was initially generated for each interview, in its entirety. As the data collection phase progressed, individual questions were coded collectively. This process allowed transparency in the generation of new thoughts or concepts as data saturation was considered. Following Interview 12, no new codes were generated. Recoding and coding of data after each interview was completed ensured that the coding process was thorough and systematic.
3. **Searching for themes;** Following coding, subthemes were generated for each question by clustering codes that described a meaningful data pattern. The drafting of the relationships between the subthemes and the possibility of major themes bringing the data together to answer the research questions also began. This is what Braun and Clarke ^23^ describe as “the jigsaw puzzle effect.” The subthemes were pieced together in a thematic mapping process where they were fed into a major recurring theme across the dataset. Following this process, we identified four major themes.
4. **Reviewing potential themes;** This process involved checking the quality of the data analysis process. Some generated codes were discarded here as they were deemed too minor or did not “fit” the research area of interest. Potential major themes were re-checked to ensure that there was sufficient raw data to support it. This involved re-reading all transcripts and coded documents to ensure that the themes generated supported the aims of the research question.
5. **Defining and naming themes;** Braun and Clarke ^23^ recommended ensuring good thematic analysis by asking whether the themes had a singular focus, were not repetitive, and directly addressed the research question. The authors were satisfied that this was the case in our mapping process and that the four major themes generated told an overall story about the data, which fulfilled the research aims and objectives. A planning document was made to narratively inform the reader of the data interpretation and why the information is related to the broader research question. This also involved mapping the participants’ quotes to support this theme. The names of the four major themes were determined during the analysis.
6. **Producing the report;** this phase involved producing a “results report,” which explains to the reader what the data uncovered. This is described in the following “Results” section.

Lincoln and Guba’s (1985) four criteria for trustworthiness in qualitative research were considered in the data-analysis phase. These factors include credibility, applicability, dependability and neutrality ^24^. Credibility was apparent through the objective portrayal of participants’ views and ensuring the establishment of coherent result writing. Applicability was seen in the development of thick descriptions of participants’ responses, which portrayed the findings in the exact way they were shared. Dependability is seen in adherence to the research process; neutrality is evident in the unbiased reporting of results and in the lead researcher coming from a neutral viewpoint outside of the healthcare areas under investigation.

## Results

### Introduction

Four major themes were identified in the comprehensive data analysis phase. Each major theme was derived from commonalities and shared meanings across sub-themes. The four themes were workforce management, synergies in healthcare, progressive futuristic care, and safe practices. A visual figure is provided in each theme result paragraph to show how the subthemes contributed to the major theme finalization.

### Theme 1: Workforce Management

**Figure 1.**
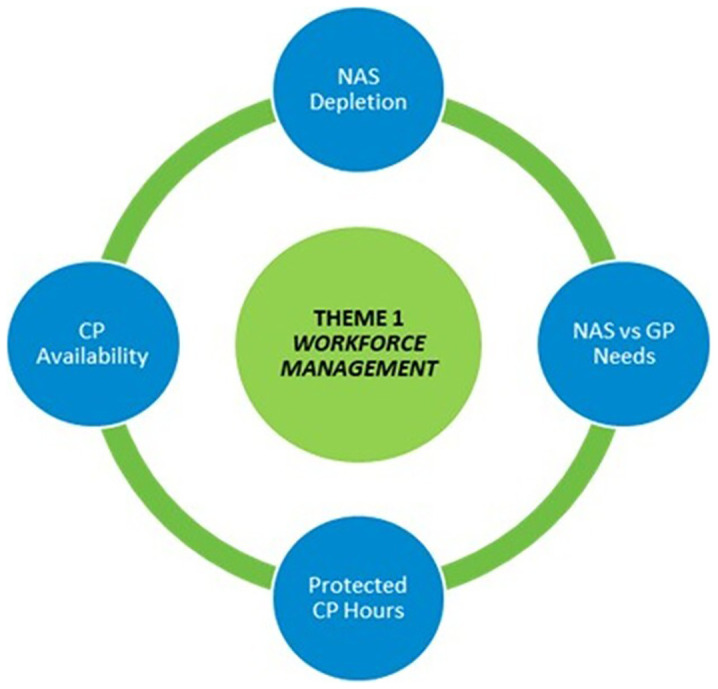
Theme One Workforce Management.

Figure 1 depicts the first theme, Workforce Management.

The positive and negative effects of CPs working with GPs in an out-of-hour setting were frequently referenced by all participants regarding workforce management. Of particular concern is the possible depletion of an already understaffed NAS. Currently, a small cohort of CPs work with GPs’ in-hours’ services. However, the CP is still employed and governed by the NAS during this working arrangement. This means that if a high-acuity call comes through the NAS and the CP is in close proximity to the call, they must cease their GP-related visit if not already with the patient and instead attend an emergency call.

*“If the ambulance service gives me a low acuity call, and a higher acuity call comes in, and I'm more proximal than any other emergency vehicle, I will be stood down from the low acuity call and dispatched to the high acuity call, and the low acuity call will wait.”* (Participant 6).

CPs voiced concerns about being unable to contribute to their role because of their NAS commitments to emergency/high-acuity calls. They unanimously agreed that for the role to be effective, the time spent with GP out-of-hours must be protected, and they must be on shift but not available to the NAS control center. They also stressed the importance of protected induction and training periods with GPs outside their NAS commitments.

*“We will not be able to work for the National Ambulance service and the out of hours service without having a cut off as to which specifically are we with at a particular point in time.”* (Participant 6).

GPs unanimously voiced concerns about the current lack of CPs and the difficulty in assigning call outs to CPs, who were then re-routed to high-acuity calls for NAS. They also agreed that unless CP time was rostered to the GP service and protected from NAS calls, the program would not be successful.

*“The National Ambulance Service has huge resource issues and will seek to have…particularly high acuity calls…answered by the nearest and most appropriate clinician, and you can see their rationale for that.”* (Participant 4)

Participants agreed that protected time for CPs would be the “ideal” when working with GP out-of-hours. However, the “reality” means that assigning CPs to GP practices in protected times would deplete an already-under-pressure NAS. The recruitment of currently experienced senior paramedics to the CP setting was also deemed detrimental to the workforce management of the NAS and its high-acuity emergency call system for patients.

*“I can see why a role like this might be attractive…That could lead to an exodus of experienced paramedics from the ambulance service, and from their…you know…what their primary role was designed to be, and that could create a problem further down the chain in a different part of the Health Service.”* (Participant 11)

*“It's all positive from a GP perspective. It's very positive from a patient perspective, but from a national ambulance perspective, there is a downside to it, and we're utilizing our scarce resource to support another service.”* (Participant 10)

Participants advised looking at the cohort of paramedics who had left the service because they could not commit to a 40-hour working week, as one solution to the possible depletion of staff from the NAS. Working part-time or mostly on weekends or nights may entice those with young families or those who can only commit part-time hours.

*“But where it might suit is the person who's given up their job because they just can't manage the 40-hr week of shift work with the ambulance service, or any of the private providers, or whoever it is, and but would be willing to do 2 evenings a week on a Saturday, or something like that. So, I suppose it's a case of looking at who can actually be employed and how can you make that work?”* (Participant 9).

Another subtheme across the data was GP worry that without experienced senior paramedics in the CP role, CP support for GP out-of-hours would not be sufficient to provide adequate “cover,” to make a difference to the GP service and their patients. GPs also voiced concerns about less senior or less experienced paramedics working with them in the out-of-hours setting, possibly outside of their scope of practice, necessitating more GP support in the long run, and making the CP service an inadequate support, and therefore more “work than help.” They stressed that autonomy and confidence in practice are key prerequisites for the CP workforce.

*“GPs have to be able to trust the community paramedic in their professional role…and I suppose, finally, the patient's got to trust all of us that we're doing the best thing for them.”* (Participant 3)

The current prerequisite for becoming a CP in Ireland is a master’s level of qualification. This would mean that all CPs would qualify as Specialist Paramedics. Some participants voiced their opinions and advised them not to recruit solely from this pool of experts. They instead suggest accepting paramedics, who have not undertaken specialist training, into the CP system with a view to undertaking the academic and professional development required for the role, while they are “on the job.”

*“I'd be slow to say that a master’s level of education is an absolute must for this to be developed in practice, and maybe we could be a bit more open and say, you could certainly participate once they're committed to undertaking additional training, be it up to master's level, I would just be slow to kind of cut off our nose despite our face. Paramedics are already very skilled professionals, which obviously gives them a great foundation when they have that additional academic training under their belt and probably gives them confidence.”* (Participant 12).

NAS versus GP needs remain a “catch-22” situation. Until paramedics’ recruitment improves in NAS, the success and potential of the CP program remains threatened. One health service manager aptly summed this point up by saying that;

“ *It will become a vicious circle, you will deplete one workforce to replete another…recruitment and retention of both GPs and paramedics, alongside training and education is the only way to solve this current problem.”* (Participant 9).

### Theme 2: Synergies in healthcare

**Figure 2.**
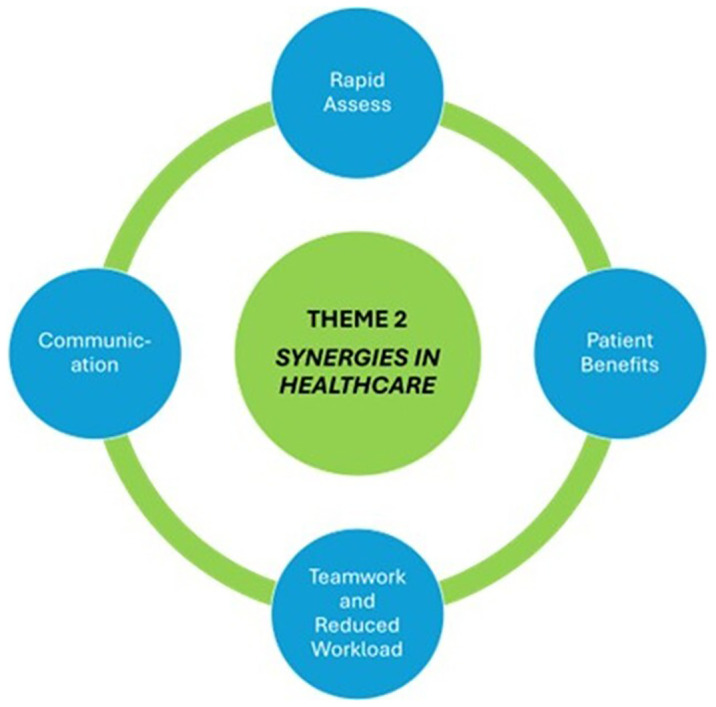
Theme Two Synergies in Healthcare.

Figure 2 depicts our second theme: synergies in healthcare.

Synergies between specialist paramedicine and GP workloads were consistently referred to by all the participants. This was deemed advantageous to the CP working within a familiar area of care in the GP out-of-hour setting. This familiarity extends to rapid assessment of patients at home. Rapid decision making about patient care is a core set of skills possessed by both GPs and paramedics. Questions can quickly be answered about the patient’s care pathway based on this initial assessment, such as Is the patient acutely and seriously unwell? Is this a non-serious injury or illness that can be managed at home? Is this a flare-up of chronic illness? Do I need external support? What diagnostics/treatments are required?

*“Being a paramedic in an ambulance service, they're used to unscheduled, undiagnosed, and undifferentiated patients for the large part.”* (Participant 11)

*“It's the higher-level thinking that comes with being a standalone practitioner. In other words, someone who will be at the scene of a patient will be able to take a really good history examination and analyse what they're seeing in front of them and be able to make a considered decision about care.”* (Participant 12).

However, one GP cautioned that novice CP may not have experienced solo response and raised the point that the scope of practice must always be a key point in assessment limitations.

*“I suppose there’s a challenge to it. They may feel like they have less support than what they would have in…in what they’ve been used to up to date. So, I think it’s…it may be more challenging than the way they work already.”* (Participant 1)

One health service manager pointed out that the “higher level of thinking” required to become an autonomous practitioner does not come from experience alone, and must be supported by an advanced level of education.

*“But certainly the key thing about you know the training that they receive is their ability to perform at a higher level of thinking. And I think that's really what distinguishes them from another practitioner… no doubt it's kind of…an honours level 8 or a level 9 qualification is where I see this sitting*.” (Participant 11).

Interestingly, a GP spoke about how paramedics usually work within strict guidelines and protocols, and when in an autonomous role as a CP, these strict guidelines may sometimes need to be considered outside of if the practitioner has a “gut feeling” or the patient complaints cause concern. (Participant 4)

*“I think the paramedics sometimes are bound to follow the guidelines and the protocols that they have and maybe left with less kind of freedom to go by a feeling, a gut feeling, or a sense that a patient isn't right. Clinical autonomy is so important…there's certain things you must transfer if X, Y and Z has happened, but it mustn't be the case that you cannot transfer, because they don't meet X, Y and Z criteria absolutely. If the patient isn't right or you have a bad feeling, or the patient is insistent that they're not right, then it should be within the CP to say, yes, okay, we will arrange transfer.”* (Participant 4)

Utilizing CP skills and experience in GP practice is an advantage that all the GPs interviewed welcomed, particularly when synergies in healthcare and timely patient care were considered.

*“So, it brings, you know, an added service to be utilized by the GP…or his or her patients. It may allow quicker access to an assessment...and potentially then…care as a result of that assessment. So, I suppose, for the GP's point of view, it would, in my view, at least, anyway…open up assessments of people by a trained professional and at an earlier opportunity. We're not always waiting for the next available slot in our diary to see someone. Somebody can be seen, perhaps by the community paramedic to do that assessment.”* (Participant 3)

Synergy in healthcare was also voiced by the CPs. They spoke of the skills and intuition required to encompass social care issues in domiciliary settings, as well as acute episodes of caregiving. Thus, it provides a more holistic episode of care for the patient and enables a referral pathway (such as social prescribing) outside hospitalization or ED attendance.

*“And we're clued into things like…we pick up on social things, maybe more than an out of hours doctor, who wouldn't really be that familiar with the prehospital or the out of hospital setting, you know, going into people's houses, and we pick up on things that might need improvement. Or you know, we are just…we're a bit more intuitive, maybe, than people who don't have the experience outside of hospitals.”* (Participant 5)

The data revealed that communication with teams and working as part of a team are vital for ensuring the success of the CP program. This ensures synergy in health care teams as a holistic unit that works well together for the good of the patient.

*“As CPs, we need to explain why we're there. We're there to fill gaps that can't be filled in other or by other people at the moment, more than likely, and we're not there to take away anybody's jobs or anybody's skills or autonomy, or whatever it is. You know, we're there to enhance what's currently available, and hopefully make it a better experience for the patient and staff, you know, because it might ease the pressure, and I think generally we get accepted very quickly.”* (Participant 6).

Most participants spoke about patient benefits when considering timely access to care that would otherwise have been inaccessible or had a lengthy wait period. This shared care or ease of access to care is a good example of a healthcare team that works synergistically.

*“So, the workforce is certainly challenged in terms of its capacity and its ability to always respond in the most timely manner to the requests of it. So, I think one of the likely, the most significant advantages is improved access to a clinician of some description, and therefore a…a faster experience of access to care in some form, and for me, that's sort of the biggest benefit in that sense…that actually providing a speedy access to a clinician and a clinical assessment than is possible at the moment with the current GP out of hours workforce.”* (Participant 10)

There was an overall positivity towards the acceptance of patients towards the CP service versus ED attendance or a GP-OOH visit.

*“Paramedics, I think, are really like a chameleon, and they have to adjust their surroundings to be accepted because so much of their work is in people's homes. So I think they of many healthcare professionals have very well-honed interpersonal skills and situational awareness and intuition to be able to adjust their manner to the patients and the families that they see. So I think those 2 things are probably really advantageous for patients.”* (Participant 7).

Synergy in healthcare is an overall consideration when GPs and CPs have the opportunity to work together to both reduce the GP-OOH workload and reduce emergency callouts to convey patients to the ED. Both groups could use core skills to manage patient care episodes and maintain safe domiciliary care. This teamwork has the potential to improve the patient experience and provide safe and effective care in the community.

“ *In the region that I work in here is very…we've very strong synergies between us [CPs], our primary care colleagues, GP, and then the broader services. So like, really, where we're operating as one team. And I suppose the utilization of individual skill sets and competencies to their highest threshold certainly is you know…it's an enabler, I suppose, in terms of maximizing the services that we have”* (Participant 10).

GPs spoke about how CPs need to be seen as a valuable resource for GP-OOH treatment centers as well as domiciliary visitors on “callouts.”

*“They should actually be dealing with patients in the treatment centres as well, and not just in the domiciliary setting because there'd be an awful lot of crossovers of knowledge based on their experience.”* (Participant 2).

*“GPs need to be clear about the professionalism and capacity of CPs to work independently and guide a care episode competently and holistically, not just as a “runner” out to homes as a middleman.”* (Participant 1)

All participants agreed that for this synergistic teamwork of interdisciplinary services in patient care episodes to be successful, the biggest obstacle would be patient acceptance of a CP consultation versus a GP or ED visit.

*“From my experience the role is generally accepted when it's understood. When the public understand that it is part of a multiprofessional approach that's based around trying to improve access for them, and that it is supported, and there are escalation channels and review channels, then, in general, that kind of presumption that this will give you better timely access to a clinician as opposed to sitting there, not seeing any clinician, then that's generally understood.”* (Participant 9).

*“A media campaign, because it needs to be socialized, doesn’t it? And so something I heard on the radio a little while ago was about how the 111 service operates in England, and it says, if you phone 111 for a minor illness, a GP, a paramedic or a nurse might answer the phone, and they will assess you. And it's just a really short radio advert that straight away, it socialises me to the idea that the GP is not the only person I might speak to. There's 2 other people, and all of those people seem reasonable to me, because a very soft voice on the radio is telling me so.”* (Participant 10)

### Theme 3: Progressive futuristic care

**Figure 3.**
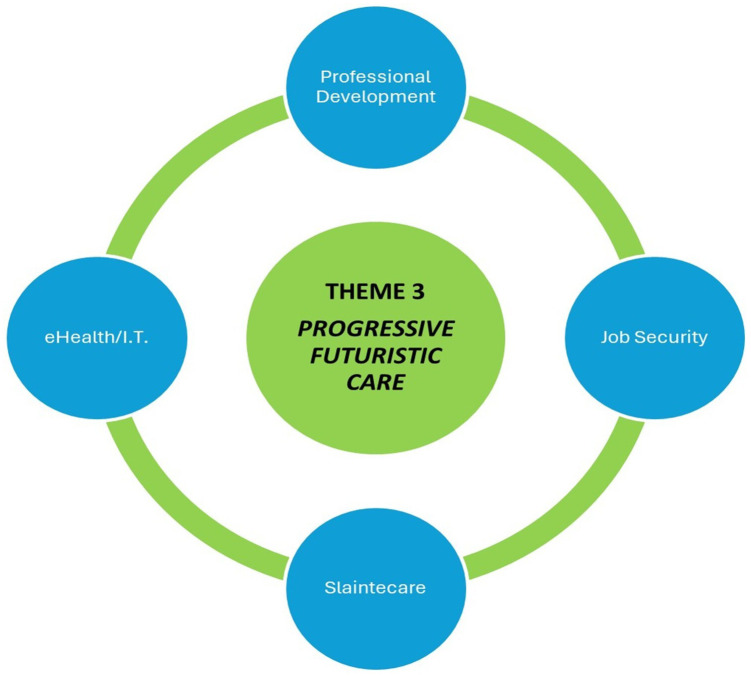
Theme Three Progressive Futuristic Care.

Figure 3 depicts the third theme: progressive futuristic care.

Most participants mentioned opportunities to provide progressive futuristic care. This included new opportunities for paramedics to pursue professional development, embracing new scopes of practice and new arenas of practice within their roles. However, CPs were also cautious and spoke of the need to be assured of job security within the GP-OOH practice, or protected CP hours within the NAS. Progressive care incorporating Slaintecare aims has also been discussed, as has the opportunity to work with advances in eHealth and futuristic IT systems.

The progressive growth of paramedics in Ireland was discussed by a health service manager who encouraged the continuing professional development of this skilled and experienced cohort of healthcare professionals.

*“When you think of how they've evolved. It's fairly phenomenal, you know, in terms of the rapid cars, the advanced paramedics, you know the various different specialties within the teams. I imagine that, like any other discipline, that they will absolutely want to work to their full autonomy of their competencies and their training, and I think that they certainly would like that autonomy.”* (Participant 12).

However, GPs cautioned that paramedics coming into the CP role would have to become accustomed and skilled at non-acute presentations in addition to the acute patient presentations they normally deal with.

*“But it's not a blue light collapse. So it's not somebody that's unconscious having a seizure. It's somebody who has weak legs, they can't stand up today, and they're running a temperature. They're going to be the kind of GP type presentations that we would hope that they will be seeing, and that they will become skilled at.”* (Participant 2).

Healthcare managers iterated that CP would need to progress in their autonomy and learn to work outside of a team setting but within their scope of practice.

*“CPs need to be registered, regulated, autonomous, healthcare professionals operating under their own registration with some conditions and presentations that they can assess and manage wholly within their own scope of practice.”* (Participant 8).

Interestingly, CPs indicated that they are already realizing that they can fulfil the autonomy required in practice, but only if they are under the umbrella of CP and not in the standard paramedic role.

*“I did 4 calls last night. I took 3 of them to hospital, one not. But if I was in my role as a community paramedic, I was in my role as an advanced paramedic last night on an emergency ambulance, I could have left those 3 people at home.”* (Participant 6).

Job security was a recurring theme amongst CPs.

*“It might be a concern of mine going into a private GP practice and resigning from that mutual service would be if that practice ceases to exist.”* (Participant 5)

The GPs questioned who would take responsibility for and indemnify CP when working within their practices. If this progressive collaboration is to be successful, all these issues must be clarified before CP professionals can be rolled out further in community settings.

*“But if the CP is working individually in a GP's practice like who's going to insure them? Are they on my insurance?”* (Participant 1)

The transformation of healthcare systems in Ireland towards models of community-based care integration is an ongoing national challenge. Health service managers mentioned the slaint-care strategy, particularly during interviews. One of the main aims of this strategy is for people to stay healthy in their own homes and communities for longer periods by developing and improving primary and community care through the provision of integrated care. CPs working in the GP out-of-hour setting can serve as key contributing opportunities within the strategy’s aim.

*“I would be in a hundred percent agreement with the paramedics, undertaking that additional training and providing that care, which is very much aligned to Slaintecare, and as described in this research, then potentially would alleviate GPs to conduct more domiciliary home visits…”* (Participant 11)

For this transformation of the Irish healthcare system to be successful, it was apparent that the participants were aware that regulations would need to change, particularly at the government and national Department of Health levels.

*“Fundamentally it allows for our patients to be treated closer to home, if not in the home. And I suppose on a governmental level, you know, this is one of the bases of Slaintecare. Let's deliver the most appropriate care as close to the patient's home as possible. So, I suppose it ticks a box for that particular aspect.”* (Participant 9)

Health service managers currently speak about the low number of GPs in Ireland, and how recruiting GPs is an ongoing struggle. They iterated that CPs have the potential to reduce the GP workload, which may entice new recruits into the sector when they see that they will be more supported in their role.

*“I suppose the potential for GPs new recruits and the graduates to go into general practice when they see that, you know they're not operating as sole traders for absolutely being the front door for everything, that there are other staff there that are, you know, rowing in to assist where they can. I think that would be really helpful. To be honest, you know that it's demonstrating that we are operating as one team, one approach going forward as a health service, and that GPs aren't a separate commodity on their own struggling with massive demand, hitting their doors.”* (Participant 2).

eHealth is an evolving era of healthcare internationally and has the potential to improve patient care episodes through the use of electronic health records (EHR), IT-based diagnostic and interface systems, and telemedicine consultations. CPs will need the support of GPs remotely in some cases, and there is an opportunity to integrate futuristic eHealth systems into the GP-OOH setting. The use of EHR is evolving in Ireland, with some hospitals now fully integrated and no longer using hard copies of patient information. For CP in a remote setting, the use of an EHR is invaluable, as they can see patient records and medical histories at a glance.

*“Patient data, and these unique identifier numbers will be of major benefit to us. If we could look up a patient history prior to us going out because a heart rate of 60 for somebody might be abnormal, where their heart rate should be 90 or should be 40, whatever it should be. You know, we don't know people's baselines going out as community paramedics, and that's a big drawback.”* (Participant 6).

All participants agreed that electronic health records would be important in the future when treating patients in community settings.

*“The missing link in that is that they don't have access to the patient's list of regular medications and their allergies and things. And that can be quite a bit different from what the patient presents them with.”* (Participant 3)

One health service manager discussed the possibility of healthcare becoming futuristic enough that a non-skilled individual could do the callout for the NAS or the GP-OOH and feed the information back via telemedicine in a triage system.

*“If you're using a kind of more telemedicine approach, you can have somebody with less skills do the actual physical assessments…and, you know, get the vital signs and all that. You know, they can be trained to that level…already are probably, and then, in conjunction with a telemedicine kind of model, have the doctor or CP make the decisions from afar.”* (Participant 8).

Virtual triage is already in operation under Slaintecare aims and objectives in some regions.

*“We've recently established an urgent virtual care service here in the region that's led out of the acute hospitals that's overseen by senior clinicians, such as consultants and emergency medicine and geriatrician, etc. And the referrals are in via GP and paramedics. But like we're already finding, you know, huge benefits from that whereby pathfinders and paramedics are being redeployed out to particular individuals on foot of the virtual triage to be able to be cared for at home. I've witnessed, you know, late eighties, 88 year old gentleman being at home, triaged in his sitting room, and you know both him and his family absolutely delighted, as opposed to coming in and sitting on a, you know, potentially a hard chair in ED, and waiting, you know, for a protracted period of time, because of the busy environments that are there.”* (Participant 11)

Participants were questioned about the use of IT-based systems when CP was working in the community autonomously. All of them agreed that this was an important factor to consider. This could enable GP engagement through video consultation if required, GP visualization of an injury or the exacerbation of a chronic illness in a patient known to them, or for the CP to question the GP about a past history or prescriptions for the patient. Health service managers spoke about the progression of diagnostics in domiciliary settings such as mobile radiology units.

*“I mean, I think, as a point of principle, people who are expected to manage clinical consultations in the out of hours setting should have access, ready access, to all of the relevant clinical information that they need to make appropriate clinical choices. I think that's a baseline kind of principle, really, that applies across the board to every professional working in that setting, regardless of their registered background. So yes, I think there needs to be clear, ready, and functioning access to the records. They need to be able to access the right clinical information to make the decision, and they also need to have a ready channel back to somebody who can help them interpret that information if it happens to take them out of their scope of practice.”* (Participant 9).

### Theme 4: Safe practice

**Figure 4.**
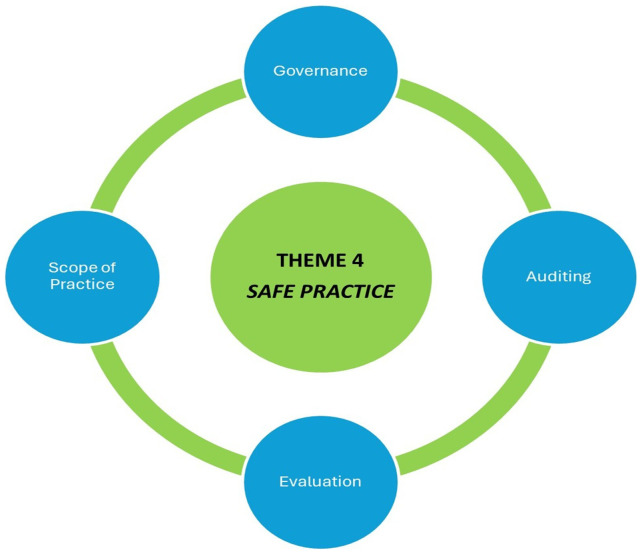
Theme Four Safe Practice.

Figure 4 depicts the fourth theme of safe practice.

The final major theme apparent in the data analysis was the importance of safe practices for patients. This involved the governance of practices, an audit and evaluation process, and CP within a defined scope. All the participants spoke about the importance of clarity in the governance of care within the CP role. Will they be governed by NAS? Will they be governed by GP practises? Would the care provided be “algorithm-driven,” as is typical of paramedicine? Would they be insured or covered by a union if there are errors in care, misdiagnosis, or any other issues of concern? These issues were raised and flagged by participants as important to decipher before work within GP out-of-hour care could begin.

*“I think there needs to be a clear, defined scope of practice and baseline training requirement for a paramedic. Entering this role, I think there needs to be clear pathways of which patients with which particular clinical problems are the most appropriate to be seen by that group of professionals matched to their need. I think there has to be a clear and well-resourced supervision and oversight infrastructure in place, and I think there has to be a process for reviewing, auditing, learning from and developing care as it continues.”* (Participant 9).

CPs spoke about feeling uncomfortable with the current governance structures and indicated that they felt that their role was currently confused. Ultimately, they wanted to understand the first regulation of this role.

*“I think a blanket role would not be appropriate, so it would have to be a clear cost, decision making and a clear call policy as to when I'm actually working for the GP or when I'm working for the National Ambulance Service, that if I'm working for the ambulance or for the GP, that the ambulance service does not inherit any of the risk.”* (Participant 5)

Health service managers spoke about the responsibility that GP would inherit from the employment of CPs in OOH services. As lead clinicians, care is governed by these factors.

*“This is going to require significant investment in time and resource from the GPs and other experienced senior clinicians currently providing care in order to oversee it, to provide that supervision, to provide that training and development, to ultimately support the paramedics to deliver safe care, effective care, and also to help them develop professionally and develop job satisfaction for them. So, I think there are considerable resources needed to safely and appropriately supervise the care.”* (Participant 11)

The possibility of adverse clinical outcomes without appropriate and robust governance systems was discussed.

*“There is also the potential of adverse clinical outcomes, and that you know that has the potential to be very real if that supervision and oversight isn't there and isn't able to support the paramedic staff to deliver the care safely.*” (Participant 8).

Participants also discussed the importance of ongoing personal and professional development for CP to maintain an up-to-date and evidence-based practice system.

*“The CP needs to have some degree of personal responsibility that I am doing a job, and that job is or requires a certain amount of ongoing education. So, there's the personal element, too.”* (Participant 3)

Most of the participants mentioned the importance of auditing. Overall, financial savings in healthcare regions and systems were deemed the least important area of audit. Instead, patient, GP, and CP feedback in a continuous reflective cycle were deemed the most important in the auditing system. Qualitative methods of engagement have been highlighted as the most beneficial way to assess how stakeholders perceive a service.

*“You need a really rich experience, experiential sort of data.”* (Participant 7).

*“So there needs to be continual auditing that needs to be…definitely oversight, you know, in terms of, and maybe regrouping and like other than from the CP's point of view, but also from service delivery. You know. I'm a big qualitative fan rather than quantitative. I don't think it's just about numbers of outcomes, experiences matter, you know?”* (Participant 10)

Most participants mentioned their evaluation of service holism. The factors mentioned included the effect on the quality of patient outcomes, patient satisfaction, effect on GP workloads, whether the CP protected the time to see GP out-of-hour patients, whether they felt supported, and whether they were working within their scope of practice. Were significant improvements made to the hospital ED admission rates and NAS in terms of time saved from low-acuity calls? Were there any financial savings from using this service? How can these services be improved? What is working well and what needs more work going forward? Is NAS under significant pressure due to workforce loss?

*“So, I think the key thing is, are they getting the appropriate care in the appropriate place by the appropriate person. And however the evaluation is structured, I think that's the question that has to be answered. How has it enabled the GP practice to function? But also, what has been the effect on other aspects of the health service, specifically the ambulance service in terms of reducing demand, and emergency departments in terms of reducing demands and subsequent knock-on hospital admissions.”* (Participant 10)

It was the GPs in the majority who voiced concern about the knowledge, experience, and educational background of the CP they would be working with. A master’s or level 9 qualification was deemed the minimum entry point for a CP who would be working autonomously in patient care in the community. The GPs also spoke about their experience within the NAS and with them in a workplace placement, giving them confidence in the CPs’ abilities and skills of triage and assessment. CPs who knew that they were working within their scope of practice and what to do if they found themselves outside of it were major factors in patient safety as they continued personal and professional development.

*“In terms of clinical skills, I think, with the right supports and training and experience and exposure, particularly guided by general practitioner, in other words, working with them on a placement basis, and I think they would be well equipped to provide that out of hospital, out of hours care in a safe way, which I think is ultimately the goal.”* (Participant 5).

One of the challenges highlighted by the participants was that the advanced training necessary to bridge the gap between true acute and emergency care and urgent primary care is broad and complex. This means that the scope of practice may continually change for CP and CPD is extremely important for patient safety.

*“So, one of the major challenges is working out what a particular training course qualification, or indeed professional descriptor actually means, in terms of what competencies come along with that. I think in the management of medical complexity, multimorbidity and interacting simultaneous health care problems, there is definitely a training need in that. I think there are needs in balancing medical and psychosocial considerations. They're perhaps not so well covered or well developed in the current scope of community paramedic practice.”* (Participant 10)

*“Some mental health problems, obstetric, gynaecological, pregnancy and sexual health related problems, infectious diseases other than the very sort of common acute ones, are all some such examples, but I think complexity and frailty and multimorbidity, and the interaction with mental and psychosocial health are the main areas of training need development.”* (Participant 7).

*“I would draw a distinction between training and ongoing professional supervision and professional development, as I think they're 2 different things. So, I think with respect to training, I think there does need to be a degree of structured centralized training against a blueprinted curriculum that's probably delivered centrally by an education provider, a higher education provider. There's also the second aspect of training, which is, you know, the more on the job day to day professional training, development, and supervision.”* (Participant 3)

Overall, CPs highlighted that working within their scope of practice was a personal responsibility and a principal factor in the safe delivery of patient care.

*“Listen to your experience and your training, and there's no ego. There's no ego in healthcare. If you don't know or you're not sure and at all concerned, you escalate it. There has to be an area, or you know, if you're just not sure that you have, you seek help, you know where you seek further advice, because even my GPs will say to me that you know sometimes, they're not sure. But look, let’s check it out, you know. Not everybody knows everything* (Participant 10)

### Inventory of pilot model considerations

This qualitative research phase provided key pilot model considerations that could be taken forward to a steering group in collaboration with the key stakeholders involved in this investigative study. Developments are informed by the work of previous authors in this area ^16^, and it is in conjunction with this expertise and new knowledge pertaining to the Irish setting that the next phase of the model development can begin. Following data analysis and results writing, Table 2 outlines what has emerged as important to the proposed pilot Irish Model of GP and CP Collaboration in the OOH Setting.

**Table 2.** Pilot Model Considerations.

NUMBER	CONSIDERATION
**1.**	Workforce Planning (NAS/GP/Protected Time/Acute Calls)
**2.**	Baseline Training Requirements-Education
**3.**	A Clearly Described but Flexible and Evolutionary Scope of Practice and Role (dependent on community needs).
**4.**	Regulation and Indemnity (currently no CP strand on the paramedic register)
**5.**	Development of Legislation Governing the CP Role (are independent licences required?)
**6.**	CPD (protected time, exams, and assessments on entry?)
**7.**	Clear Patient Pathways (matching the right HCP to their needs)
**8.**	Governance (well-resourced supervision and oversight infrastructure including PHECC, GP, ED Consultants, NAS and DoH representatives)
**9.**	Robust Audit and Evaluation System (process for review, audit, learning from and developing care as the pilot model continues)
**10.**	Department of Health Collaboration and Involvement (at all levels)
**11.**	Clear Communication Pathways Between MDTS (physio, dietician, PHN, ANP etc)
**12.**	Communication Pathways for Patients and the General Public
**13.**	Rural vs Urban Considerations (no one size fits all, geographical aspects)
**14.**	Funding and Financing
**15.**	IT and eHealth Infrastructure Training and Design

## Discussion

### Theme One

Workforce management is a recurring subtheme in the qualitative data collected. Both health service leaders and CPs spoke about the possible depletion of an already under pressure NAS if paramedics were to leave the emergency service and fulfil CP positions instead. The current work of CPs “in-hours” sees them attend low acuity calls for GPs, but they also have to be on call for high acuity calls in proximity to their location. Both CPs and GPs would like to see CPs working in protected capacity, whereby they cannot be contacted by the NAS for emergency calls when on duty with the GP. GPs stress that this is the only way the proposed GP-OOH collaboration will work for them and their patients. On the other hand this “depletion” of emergency paramedical personnel, as they work with GPs OOH, may actually defuse and triage possible 999 callouts, thus negating the necessity of patients calling the NAS in the first place.

An Oireachtas Irish Government Report ^25^ in 2023 highlighted significant shortfalls within the Irish NAS despite tireless effort and increased demand on frontline and operational paramedics. A HSE Report ^26^ in 2024 set a target that 80% of life-threatening incidents should have an ambulance response within 19 min. In 2023, the average response time was 27 min. By 2023, NAS in Ireland had a workforce of approximately 2,000 paramedics, with a strategic plan to increase this workforce to 4,000 by the end of 2026 ^26^. Considering these figures, it is not surprising that health service managers, in particular, voiced concern about NAS depletion in the GP-OOH setting in a CP capacity. However, it must be considered that the GP workforce in Ireland is also under continued strain. There are currently only 0.86 GPs per 1,000 people working in Ireland, with approximately 33% of this cohort either entering retirement or likely to retire within the next 10 years ^8^. The HSE National Service Plan (2025) ^2^ reports an increased life expectancy of approximately 82 years, with a growing need for an aging population requiring expanded and strengthened primary and community care services. Pressures on the GP and primary care systems nationally will also be exacerbated by the launch of an “Urgent Care and Operational Plan” ^3^ by the HSE. This plan aims to improve timely and safe access to services for Irish patients in the community, which strives to reduce stress in the ED and acute hospital services. It is therefore timely that an evolution in current services be considered, particularly within paramedic and GP settings.

The CPs interviewed in this study highlighted that the high-acuity 999 system in Ireland could currently be experiencing excess demand driven by calls from patients who cannot access primary care in a timely manner, particularly in the OOH setting. One CP spoke about how, during four emergency calls in a nightshift, if he was in a CP role, three of those patients could have been treated at home, with just one requiring genuine ED acute care. However, in the role of paramedics for NAS, and not a CP working in collaboration with GP- OOH, all these patients had to be conveyed to the ED instead. GPs also spoke about the poor recruitment and retention of newly qualified GPs in Ireland and how this could be addressed through collaboration of care with CPs, whereby new recruits saw a reduction in stress and workload, thus encouraging them to remain and work within the Irish healthcare system. The only way to measure whether this collaboration of care can affect change across the Irish healthcare system, where depletion of the NAS will replete an understaffed GP-OOH system, and in the process reduces 999 calls to the NAS, thus reducing the burden on ED departments, is to run a fully staffed pilot program. Measuring its effects across the healthcare system through mixed methods of evaluation in continuous reflective cycles is extremely important to maximize feedback and evaluation of the service. However, full staffing of the program will be a challenge, as paramedics interested in pursuing CP qualifications may struggle to fit studying and placements in full time jobs ^27^. Another factor to consider is the competition for further education within the NAS workforce, thus causing unrest and possible retention problems within the service. Another alternative is that CPs work for/with GPs as employees and are funded by the Department of Health. This ensures that CPs work independently in GP practice. However, the NAS could, therefore, train paramedics with a view to employment in the NAS, who would then leave to work for GPs when qualified. These factors must be considered by stakeholder groups prior to the finalization and launch of the proposed model. A detailed employment and recruitment plan must be carefully considered before a pilot can become a reality.

### Theme 2

The potential for synergy in the Irish healthcare system was a consistent theme in the reviewed qualitative data. The Oxford Dictionary defines synergy as “the interaction or cooperation of two or more organisations to produce a combined effect greater than the sum of their separate effects” ^28^. This was first conveyed by participants when they spoke about the rapid assessment skills possessed by both the GPs and CPs. Patients can avail triage in either the domiciliary or GP setting, and it can be deciphered whether the presentation is acute or non-acute, if external support is required, and if diagnostics and treatment are needed. This collaboration of care will have immediate benefits for patients who can receive timely care in their own homes, and will minimize unnecessary conveyance to acute care settings through NAS 999 calls to already overburdened EDs. However, clear communication pathways will need to be forged so that the patient is satisfied with the service, safe and timely care is provided, and they fully understand the role of the CP.

Collaboration between CPs and GPs has the potential to benefit both the NAS emergency call system and decrease the workload of GPs. However, another important consideration is that CPs can also immediately contribute to expertise in emergency situations that are present in the GP-OOH setting. This is another synergistic factor to be considered and is an important factor for integration into the proposed pilot model. There is potential for interdisciplinary roles within the GP-OOH setting to work together to develop new skills and learn from each other. Current CPs spoke about the lack of communication in the current “in-hours” CP pilot in Ireland among differing primary care teams. They spoke of unrest from practice nurses, and GP drivers in particular. Recent studies conducted in Ireland have reported that a lack of professional boundaries in primary care settings can cause workplace tension owing to an overlap in clinical responsibilities ^27^. Therefore, it is important to include communication in the proposed model as a major factor in its development. Stakeholders from key positions in the GP-OOH setting must be well informed of the role of the CP. Working together to enhance one another’s skills can be a key diffuser in workplace tension. Emergency care and rapid diagnostic skills can be offered by the CP, with chronic symptom management, medications, and diagnostic primary care testing offered by GP and practice nurses. An important consideration was mentioned by a UK study’s findings in 2019 ^17^, which cautioned that the mentorship required from GPs may cause barriers when a new trainee is introduced into the general setting. It will require considerable time and training within work placements with CPs before the role is seen to be of benefit within GP-OOH practice. However, the long-term trajectory of a service must be considered and appropriately communicated. Although the GP participants in this study spoke about the CP programme possibly being “more work than help,” they also spoke about the possible positive evolution of the service once a CP was integrated within the team, making a difference to safe, timely patient care. This synergistic teamwork is also welcomed by GPs within the practice itself, where CPs are not just considered in their traditional “mobile healthcare” role, but as integrated healthcare professionals within the practice itself.

### Theme 3

The progression of the paramedic role in Ireland offers new opportunities for paramedics to pursue professional development and embrace a new scope of practice within their basic qualifications. Individuals currently begin their careers as students with NAS, upon which, when their first year is complete, they register as postgraduate paramedic interns with PHECC ^29^. After the second year, students register as paramedic practitioners, where responsibilities include the care, treatment, and movement of patients while maintaining their own safety, equipment, and ambulance vehicles. The completion of the third year allows paramedics to graduate with a BSc level eight degree, respectively. After five years of experience as a paramedic, they may apply to the Specialist Paramedic-Community Paramedic MSc. Programme. Alternatively, a minimum of five years of practical experience and BSc. (Hons) degree or equivalent, applications can then be made to the advanced paramedic program with a focus on advanced patient care. It is this level of training that GPs, in particular, perceive as a minimum entry into the CP education program currently being offered in Ireland. Professional fulfilment through the development of skill sets within a structured environment may invite paramedics. This evolving role has expanded to other countries as well. In the UK, paramedics can expand their education to include more autonomous roles by prescribing rights ^17^. The possibility of these emerging roles within the Irish healthcare system affords the opportunity for synergy within the national healthcare system to continue functioning in support of Slaintecare’s aims. This encourages the recruitment and retention of paramedics in the NAS as new exciting career opportunities begin to evolve. This evolution of the paramedic role should also have a knock-on effect in the GP system in Ireland, as new GP recruits are less burdened in their roles in practice, thus encouraging their retention. The use of paramedic skillsets within the GP-OOH setting as semi-autonomous, skilled professionals should also lessen the burden on the ED setting in Ireland as cohorts of non-acute patients are treated comprehensively in their own homes. This leads to the progression of Slaintecare in Ireland, as primary and domiciliary care of patients is promoted, thus lessening the burden on acute and sub-acute hospital settings ^6, 28^.

Considering the patient’s perspective on this new progression of CP care is particularly important. If a patient does not understand or accept the role of the CP in the GP-OOH setting, they will still revert to a 999 call to the NAS, particularly if the GP-OOH system has significant delays in providing appointments. A study in the US reported patient confusion regarding the paramedic role in the GP setting ^30^. A research participant from the UK advised on a media campaign in Ireland prior to the launch of the CP in the GP-OOH setting. Newspaper and social media portals can convey messages to the public, reducing confusion when a CP instead of a GP attends to their OOH medical needs. Further published literature suggests that the majority of patients are satisfied with being treated by paramedics in the general practice setting and in the home setting and did not find delayed treatment. However, they preferred GP visits when problems were severe or complicated ^31, 32^. Overcoming this problem was mentioned by CPs in our study when they spoke about the importance of using IT and eHealth systems in their consults, particularly when a GP opinion or patient reassurance from their GP was sought.

The Irish Slaintecare report, 2025 ^33^ dedicated capital to Technology and Transformation across the Irish health service. This incorporates the implementation of a Digital Health Implementation Roadmap to establish a pathway for the use of digital technology within the healthcare system to create a patient-centered, digitally enabled health and social care setting. The research participants in our study, in particular CPs, stated the importance of digital systems in their work in the GP-OOH setting. This primarily incorporates access to an EHR for each patient and an IT interface to enable collaboration and GP oversight in areas outside their scope of practice. The European Commission ^34^ is presently targeting initiatives to encourage the adoption of eHealth platforms in the EU. eHealth can be described as any form of IT that can connect providers, patients, and governments to improve health, well-being, and healthcare provision ^35^. Therefore, this opportunity for CPs to collaborate with GPs outside practice will contribute to the evaluation of technologies in the primary care setting. This can occur in the form of EHRs, diagnostic scans, X-rays, and virtual GP consultations in the patient’s home, without the need for physical attendance.

Consideration of the use of eHealth systems in CP continuing education is also apparent in literature. eHealth platforms designed specifically for CPs can allow for continuous education, regardless of where or when an individual’s study would take place ^36^. This could also be used when a paramedic who has not yet completed their advanced or CP training is accepted into the primary care setting to “learn on the job,” as suggested by one of our participants. This form of “internship” would lessen the depletion of the current workforce of advanced paramedics from the NAS and encourages a form of education that is enriched with workplace insight and scientific theory. The literature also encourages eHealth systems in healthcare settings as a form of networking between other practices and facilitates the sharing of results ^37^. CPs and GPs in the OOH setting can therefore have a shared portal of knowledge, where the successes and pitfalls of the pilot model are discussed, explained, and evaluated on a reflective, continuous cycle. Although IT and eHealth infrastructure are currently evolving and becoming more commonplace in healthcare, it is important to remember that not all individuals will be confident and competent in its use ^38^. Therefore, a training module in such systems needs to be part of the CP and GP education plans.

### Theme 4

Safety practices were a recurring theme in this study’s reviewed data. Participants spoke about clinical governance, the importance of audits and evaluations, and in particular, the CP scope of practice. Clinical governance across healthcare is a system that ensures that healthcare organizations and healthcare professionals (HCPs) are accountable for the delivery of safe, effective, comprehensive care ^39^. This involves policies and procedures that guide patient care management in health care settings. Clinical governance is essential for maintaining patient safety and protecting health care professionals in the delivery of effective patient care. Indemnity in health care ensures HCP protection during malpractice claims or negligence ^40^. Currently, in Ireland, paramedics are indemnified by the State Claims Agency through the Clinical Indemnity Scheme when employed by the HSE ^40^. Most GPs in Ireland have private indemnity covers but are covered by the State Claims Agency when they work for the HSE. CPs in our study voiced concerns about indemnity when providing a callout for a GP in a primary care setting. If this GP has a private indemnity, it may not cover CP if there are patient claims of malpractice during the care period. This is an area of the pilot model that needs to be transparent if the recruitment of CPs to the GP-OOH setting is successful. The second aspect is the governance of CP practices. The PHECC in Ireland is an independent statutory regulator in Ireland who provides clinical practice guidelines (CPGs) that govern the scope of practise of practitioners (including paramedics and advanced paramedics) and approves organizations to implement such CPGs and therefore be able to employ practitioners ^41^. A separate ICGP Professional Competence Scheme currently governs the scope of the GPs in Ireland ^42^. If there is to be a crossover of governance, particularly within a private GP practice, where the GP provides services but works outside the HSE, then a new Department of Health in initiative leading the scope of practice guidelines for CPs in Ireland must be considered. It is also worth considering that a defined and clarified scope of practice for the role of CP in GP-OOH settings can avoid antagonism from other primary care HCPs within the service. Working as part of a collaborative team and not as “stand alone” practitioners will undoubtedly encourage effective teamwork within the general practice setting.

There is a paucity of robust evidence in the current literature to support the efficacy of community paramedics in GP-OOH settings with regard to economic, safety, morbidity, and mortality outcomes ^12^. However, there is international evidence regarding comprehensive education planning to ensure comprehensive skillsets and knowledge before a CP joins a GP-OOH setting as an autonomous practitioner. A UK based CP cautioned that the paramedic role within a GP setting required more extensive skills and knowledge than the current paramedic training offered. This CP advised a 2-year MSc course of academic study (encompassing pathophysiology, advanced clinical examination, history taking, clinical reasoning, project management, and clinical setting supervision with a designated medical mentor) ^43^. Existing literature suggests that the core capabilities of paramedics may not on their own be sufficient to manage patients with chronic disease and multimorbidities ^15^, and a further study cautioned that uncertainty regarding core competencies required by paramedics in the GP-OOH setting can serve as a barrier to its success ^44^. The same UK study described a program in which paramedics underwent 450 h of advanced training provided by ANPs and GPs with a competency framework and job proficiency grading before participation was granted in GP-OOH services ^44^. Another recent UK study produced a series of practice implementation recommendations for paramedic placement in primary care ^45^. The recommendations of these recent studies can be adapted for CPs in the Irish setting. These considerations can contribute to diminishing the concern for the CP scope of practice voiced by GPs in our study. It is important to note here that the CP participants in our research study were aware of “not knowing it all, all the time” and explained how they would use the GP to revert to if they felt outside their scope of practice despite education and experience levels. This demonstrates professional accountability, and portrays individual clinical governance awareness when considering the provision of safe and effective patient care.

Auditing a health service involves systematically reviewing policies, practices, and records to ensure that they meet pre-established and agreed-upon standards of care. This also ensured that changes were made to ensure safe and evidence-based patient outcomes ^46^. The evaluation of a health service follows the audit process, or can be performed in conjunction with it. Through evaluative processes, areas for improvement in program development and policy reform can begin ^47^. Important to the evaluative process are the opinions of CPs on the ground and the GPs with which they collaborate. The training and development provided can then be realistically and experientially evaluated through reflection and regular feedback from both parties. Consideration of eHealth portals for the continuous audit and evaluation of the care being provided would enable real-time adjustments to policies and procedures, as the proposed pilot model unfolds. Distance qualitative interviews through digital means can also be used, as can stakeholder meetings on a cyclical basis. Second, patient experience and outcomes need to be audited and collectively evaluated to add a voice to how patients perceive the service. This can be achieved through quantitative reviews of post-episodes of care, alongside qualitative interviews with key cohorts of patients and their families. Finally, the cost-effectiveness and downstream effects on GP face-to-face consultations, ED attendances, and 999 NAS calls should be audited and evaluated during and after the pilot phase of the model rollout.

## Conclusion

The integration of CPs into GP-OOH care in Ireland can be successfully launched if workforce planning is carefully considered. The role of CP must be defined and clearly regulated in conjunction with the NAS, the PHECC, and the Department of Health in Ireland. Patient care must be governed by safe and effective practices that are continuously audited and evaluated, and the scope of practice must be maintained by each CP through a guided education system that incorporates continuous professional development. GPs can provide support to CP as required through the use of information technology. GPs and CPs can work together to synergize healthcare and provide safe and effective care to patients in the domiciliary setting during out-of-hours, thus reducing emergency department attendance and increasing the GP’s capacity to provide face-to-face consultations. The pilot model development of this type is in line with the Irish Slaintecare Objectives, the HSE Urgent Care and Operations Plan, European Commission Objectives, and the DoH National Service Plan. This model has the potential to improve patients’ timely access to the right primary service for their needs, and to improve patient experience and satisfaction with the Irish National Health Service.

## Limitations

Currently, there is no GP-OOH or CP collaboration model in Ireland. Therefore, research participants were purposively sampled from a critical pool of experts. This may have added subjectivity to the provided information. The sample was limited to 12 participants primarily from Ireland. Therefore, the results may not be generalizable to other international contexts.

## Data Availability

No data will be made available from participant transcripts or recordings in this qualitative study. Recordings have been deleted in line with the ethics committee stipulations, and no transcripts or participant information can be shared outside the authors of this paper. This project contains extended data that is available at the data repository “Zenodo” at https://doi.org/10.5281/zenodo.17208052 ^48^ Cunningham, C. and Barry, T. (2025). Interview Schedule Rationale and Reference List for Developing a Complex Intervention to Integrate Community Paramedics in GP Out-of-Hours Care in Ireland: A Qualitative Study. https://doi.org/10.5281/zenodo.17208052 ^48^ Data are available under the terms of the Creative Commons Attribution 4.0 International license (CC-BY 4.0).
