# Supplementary material for: Developing A Complex Intervention to Integrate Community Paramedics in GP Out-of-Hours Care in Ireland: A Qualitative Study
Source: HRB Open Res. 2026 Jan 9;9:6. [Version 1] doi: 10.12688/hrbopenres.14246.1 (PMC12891948; doi:10.12688/hrbopenres.14246.1)
Supplement: Peer review history

# Reviewer report: Reviewer response for version 1

**DOI:** 10.21956/hrbopenres.15668.r53059

**Published:** 2026-02-10

**Authors:** Buck Reed

**Affiliations:** 1 Charles Sturt University, Bathurst, Australia

This is a timely piece of work as it mirrors discussions about the use of paramedics in primary care to address shortages in the GP workforce in Australia, New Zealand and other jurisdictions. 

 The article outlines the need for alternative pathways for especially unscheduled primary care in light of GP workforce and access challenges. The premise of the work is sound. 

 The methodological approach was consistent with similar qualitative research. Braun and Clarke are a well-accepted approach for this type of thematic qualitative work. The population recruited is reflective of the diversity of professionals in this space and is large enough to likely reach data saturation. The thematic analysis is sound and consistent with other literature in this space. The reporting of the result is detailed and has a good mix of analysis and quotes from participants. 

 The conclusions of the work are consistent with other literature in the space and were unsurprising the reviewer. Recommendations are pragmatic and sensible and in line with other work from other jurisdictions. This work represents an important discussion about paramedics in primary care which is being increasable recognised globally as a pragmatic and novel strategy to providing increased healthcare access and equity, especially in rural and remote populations.

Is the work clearly and accurately presented and does it cite the current literature?

Yes

If applicable, is the statistical analysis and its interpretation appropriate?

Not applicable

Are all the source data underlying the results available to ensure full reproducibility?

Yes

Is the study design appropriate and is the work technically sound?

Yes

Are the conclusions drawn adequately supported by the results?

Yes

Are sufficient details of methods and analysis provided to allow replication by others?

Yes

Reviewer Expertise:

I am a qualitative researcher with a background in phenomenology. I primarily research in paramedic workforce development and professionalsation. My PhD was in the introduction of regulation to paramedics in Australia. I am currently engaged in a project to examine the use of paramedics in primary care in rural Australia.

I confirm that I have read this submission and believe that I have an appropriate level of expertise to confirm that it is of an acceptable scientific standard.

# Reviewer report: Reviewer response for version 1

**DOI:** 10.21956/hrbopenres.15668.r53052

**Published:** 2026-01-30

**Authors:** David Long

**Affiliations:** 1 Edith Cowan University School of Medical and Health Sciences (Ringgold ID: 204605), Joondalup, Western Australia, Australia; 2 University of Southern Queensland, Ipswich, Australia

The work is timely, addressing a significant gap in Irish healthcare research, and is well-structured, progressing logically from background and rationale to methods, results, and discussion.

 The rationale for the study is well-articulated, highlighting the paucity of literature on integrated CP models and justifying the qualitative approach.

 In the spirit of enhancing the article’s impact, the authors may wish to consider the following points for a more nuanced discussion:

-  Minor errors in grammar and paragraphing should be addressed for clarity. For example, consistent use of upper/lower case in sentence case formatting and appropriate paragraph breaks for new ideas.
-  Please ensure abbreviations are used consistently throughout the manuscript. For example, ED (emergency department) and General Practitioner (GP).
-  Saturation. The discussion of a systematic review to uncover how many participants have been recruited to reach saturation is, in my view, mute. Although clarified to an extent in the Data Collection section, I would suggest that saturation is less about number of participants, but rather about whether any further areas of theoretical interest have emerged from the data.
-  Under the Data Analysis section, “each interview was electronically transcribed to a word document. The transcripts were read by CC and “corrected”’. My apologies if I missed it elsewhere, please clarify what "corrected" means?
- In the Interview Schedule ( https://doi.org/10.5281/zenodo.17208051), Part 2 states “Are you familiar with the concept of Community Paramedic and what their current role involves? Are you familiar with the GP OOH system in Ireland and what this role involves?” If the participant answers “No”, what information does the interviewer then provide? Is it possible to then influence/bias the participants responses?
- The “Data availability” statement notes that transcripts and recordings will not be made available due to ethical concerns - and certainly understandable. However, whilst confidentiality is paramount, the authors may like to consider providing a more detailed coding audit trail, like a coding tree, to show how raw data was transformed into findings.
-  For clarity (and it could just be my short memory!) please continue naming themes in the sub-titles under the Discussion section, as done in the Results section.
-  I noted the study did not include consumers/patients as participants. Their involvement could add epistemic, ethical, and pragmatic value to the research. May I recommend that future studies should consider including consumers as partners.

In summary, I concluded that with some minor grammatical edits, the article carries sufficient academic/scientific rigour to warrant indexing.

 Thank you again the opportunity to review your paper and I hope my reflections add value to your paper.

Is the work clearly and accurately presented and does it cite the current literature?

Yes

If applicable, is the statistical analysis and its interpretation appropriate?

Not applicable

Are all the source data underlying the results available to ensure full reproducibility?

Partly

Is the study design appropriate and is the work technically sound?

Yes

Are the conclusions drawn adequately supported by the results?

Yes

Are sufficient details of methods and analysis provided to allow replication by others?

Partly

Reviewer Expertise:

Qualitative research; Constructivist Grounded Theory; Community Paramedicine, workforce development

I confirm that I have read this submission and believe that I have an appropriate level of expertise to confirm that it is of an acceptable scientific standard.
